# Vagus Nerve Stimulation Therapy in Epilepsy: An Overview of Technical and Surgical Method, Patient Selection, and Treatment Outcomes

**DOI:** 10.3390/brainsci14070675

**Published:** 2024-07-02

**Authors:** Myriam Abdennadher, Pratik Rohatgi, Aneeta Saxena

**Affiliations:** 1 Neurology Department, Boston University Chobanian & Avedisian School of Medicine, Boston Medical Center, Boston, MA 02118, USA; 2 Neurosurgery Department, Boston University Chobanian & Avedisian School of Medicine, Boston Medical Center, Boston, MA 02118, USA; 3 Biogen Idec, Cambridge, MA 02142, USA

**Keywords:** seizure, vagus nerve stimulation, epilepsy, neuromodulation, surgery, epilepsy comorbidity

## Abstract

Epilepsy affects over 65 million people worldwide. One-third of people with epilepsy do not respond to medication and may benefit from surgery. Vagus nerve stimulation (VNS) is the first neuromodulation therapy for the treatment of drug-resistant epilepsy. This method is used in combination with anti-seizure medications in adults and in the pediatric population. VNS has also been demonstrated to have benefits for some epilepsy comorbidities, such as depression, and can be used in combination with other neuromodulation therapies in epilepsy. The authors present an overview of VNS physiology, patient selection, surgery and risks, neuromodulation therapy, and application to epilepsy comorbidities.

## 1. Introduction

The vagus nerve stimulator (VNS) is the first FDA-approved neuromodulation device for the treatment of drug-resistant epilepsy. VNS has evolved rapidly since early experiments by Bailey and Bremmer in 1938. Currently, VNS is a standard epilepsy surgical option and was recently approved for other neuropsychiatric conditions such as depression. This overview will focus on VNS as a treatment option for patients with epilepsy. We will present the technical components of VNS and an outline of interrogation and programming, followed by the current presumed mechanisms of action, patient selection, surgical procedure and expected complications, and the potential use of VNS for major comorbidities of epilepsy.

## 2. Background on VNS Physiology and Evidence

**Figure 1:**
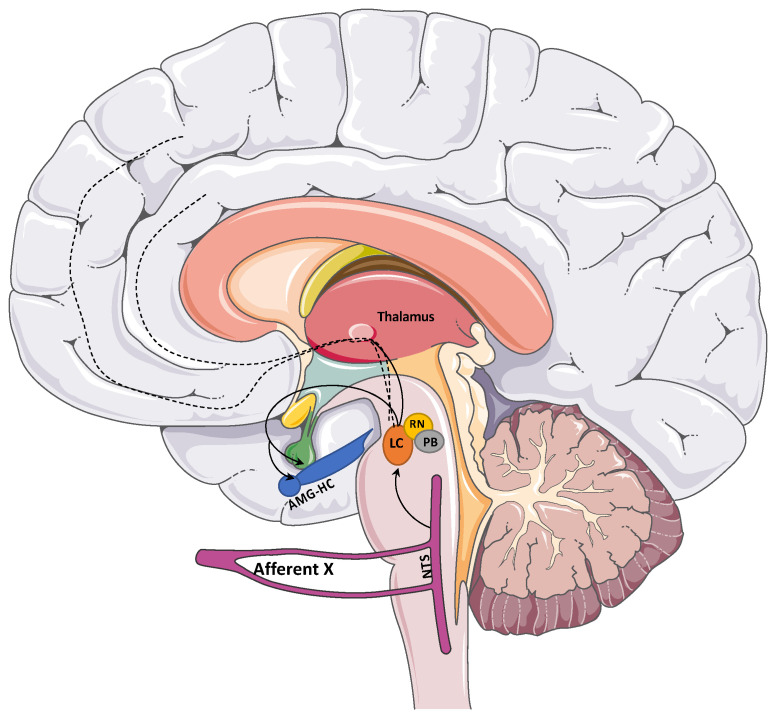
Afferent pathways of the vagus nerve. Afferent vagus nerve projects to nucleus tractus solitarius (NTS). NTS relays information to reticular formation, amygdala (AMG), hypothalamus, and forebrain through locus coeruleus (LC) and raphe nuclei (RN). HC: hippocampus, PB: parabrachial nucleus.

Although the vagus nerve is traditionally known for its efferent parasympathetic pathways to visceral organs (heart, lung, gastrointestinal…), about 80% of the vagus nerve provides afferent conduction to the brain [1,2]. VNS mechanisms for affecting conditions in the brain are not fully elucidated. Several hypotheses exist, including the following: (1) the desynchronization of electroencephalography (EEG) through the tractus solitarius and the medullary reticular formation pathway; and (2) decreased excitatory/increased inhibitory neurotransmission [3,4,5]. VNS’s central effect is generated by the stimulation of afferent unmyelinated type C fibers of the vagus nerve [2,4,5]. The afferent vagus nerve projects to the ipsilateral nucleus tractus solitarius (NTS) and the caudal portion of the contralateral NTS. The NTS relays information to the reticular formation in the medulla, forebrain limbic structures, and forebrain largely through brainstem nuclei, namely the locus coeruleus (LC) and raphe nuclei [2,6], in addition to direct projections to the amygdala and the hypothalamus [2,6,7]. Animal studies have demonstrated LC activation after transient and chronic vagus nerve stimulation [5,8] and the downstream release of norepinephrine at the amygdala, hippocampus, and prefrontal cortex [2,9,10]. The exploitation of vagus nerve physiology remains the prevailing working theory behind VNS safety and efficacy. A recent work has shown a neuroprotective effect by inhibiting pro-inflammatory cytokines and contributing to blood–brain barrier integrity, exerting a systemic and local result on neurological disorders and multiple organs (gut microbiota, cardiovascular) [11]. Other studies have found a VNS-induced theta rhythm with underlying gap junction functional changes in astrocytes, and a potential effect on local excitability at hippocampal slices [12,13,14]; this suggests a possible other mechanism of VNS neuromodulation. Figure 1 illustrates a schematic representation of the anatomical central projections of the vagus nerve.

## 3. Overview of VNS Safety and Efficacy

Five clinical trials (E01 through E05) were conducted in the US and other countries (VNS Therapy System, Epilepsy Physician’s Manual, LivaNova, US Version, April 2021) (hereinafter Physician’s Manual) (Section 2—Epilepsy Information—Clinical Studies) [15] and demonstrated efficacy in focal drug-resistant epilepsy [16,17,18,19]. In those trials, nine deaths occurred, including four due to sudden unexpected death in epilepsy (SUDEP) [17]. Two studies were randomized, blinded, active control trials: E03 enrolled 114 patients from twelve sites in the US, two in Germany, and one in each of Canada, Holland, and Sweden [16]; E05 enrolled 254 patients in the US. Participants were assigned therapy in the following two groups: high stimulation (treatment) and low stimulation (control) in patients with six or more focal impaired awareness seizures per 30-day period. The results showed short- and long-term treatment responses [16,20]. E03’s short-term treatments (14 weeks) showed significant differences in mean seizure reduction rate, as follows: 30.9% in the high stimulation treatment group versus 11.3% in the low stimulation control group. The responder rate (a >50% seizure reduction) was higher in the high stimulation group (at 38.7%, versus 19.4% in the low stimulation group) but did not reach statistical significance [16,21]. In 2012, Klinkenberg et al. reported the results of the first study demonstrating the safety and efficacy of VNS in 41 children, 4 to 18 years old, with drug-resistant epilepsy [22]. This study looked at the baseline (12 weeks), blinded two groups, treated them with high and low stimulation (20 weeks), and completed an add-on treatment phase (20 weeks). The most common side effects were similar to those reported in adults (voice alteration, coughing, and throat pain) and most were transient and stimulus-related. There was no significant difference when high and low stimulation groups were compared. However, seizure frequency and severity (Chalfont Seizure Severity Scale: NHS3) decreased at the end of the add-on phase, where both groups received high stimulation [22]. Long-term results (16–18 months) showed significant seizure reduction (52%) in the high-stimulation group compared to the baseline [20]. A responder rate of 31% in the high stimulation group versus 13% in the control group was also reported without statistical significance (George 1995) [20]. Similar findings were seen in E05: there was a 27.9% seizure reduction in the high-stimulation group versus 15.2% in the low-stimulation group [18], with significant seizure frequency reductions. This study also demonstrated a higher global quality of life improvement in the high-stimulation group [18]. There was no significant difference in responder rate.

Several longitudinal studies showed an increment in seizure frequency reduction with duration of therapy [20,23]. A twelve-month follow-up of patients enrolled in E05 (analysis of 195 patients) showed that 35% had >50% seizure reduction and 20% had >75% seizure reduction compared to baseline. The PuLsE study (conducted at 28 sites in Europe and Canada) showed significant improvement in seizure control and quality of life in patients treated with adjunctive VNS compared to patients on medical management (best medical practice) [24]. A study conducted in Japan by Kawai et al. showed progressive improvements in median seizure reduction and responder rate (>50% seizure reduction) over the course of the first three years, reaching 66.2% and 58.8% by year three [23]. Similar results were found in a larger study by Englot et al., specifically a progressive increase in seizure freedom over time in 5554 implanted patients (with similar results shown in a review of 28 past studies involving 2869 patients) [25]. Forty-nine percent of patients responded (≥50% seizure reduction) to therapy within 4 months from implantation; 63% responded between 24 and 48 months. Evidence in controlled trials and “real life” follow-up studies supported early and progressive treatment response in patients treated with VNS, with adequate patient selection for surgical success.

## 4. Patient Selection

Adequate candidate selection is important for treatment success and to avoid invasive therapy for those who may not benefit from it. Initially indicated and designed for people with focal epilepsy, VNS now is also indicated in generalized epilepsy. Pivotal trials focused on patients with focal epilepsy, but more recent studies showed benefits in people with generalized epilepsy [26,27,28] and Lennox–Gastaut Syndrome (LGS) [29,30]. Patients with LGS were significantly more likely to achieve a >50% reduction in seizure frequency with corpus callosotomy versus VNS (85.6% versus 57.6%; RR: 1.5; 95% CI: 1.1–2.1); however, VNS had lower rates of morbidity than corpus callosotomy, which can lead to disconnection syndrome (or split-brain syndrome, characterized by abnormal interhemispheric transfer of information) [31]. A meta-analysis of 480 people with LGS suggested that VNS is safe and should be considered as an adjunctive option for people with LGS [29]. Therefore, VNS represents an attractive option in LGS patients because it is less invasive [32,33]. Predictors of good treatment response after VNS therapy include the following: age at epilepsy onset (>12 years), generalized seizure type, and non-lesional epilepsy [25]. In addition to seizure reduction, VNS has shown benefits for epilepsy comorbidities, such as depression and headache [34].

VNS is U.S. Food and Drug Administration (FDA)-approved in patients 4 years and older with focal and generalized drug-resistant epilepsy. Other candidates are patients with multifocal epilepsy, generalized epilepsy, and patients with other comorbidities that may benefit from VNS [35]. Patients who refuse focal resection or other neuromodulation therapies such as responsive neurostimulation or deep brain stimulation should be offered VNS therapy. This adjunctive treatment does not require the localization of seizure focus and should be considered in patients with confirmed drug-resistant epilepsy but with an unlocalizable seizure focus [36]. The evidence of VNS efficacy in depression treatment supports VNS therapy for epilepsy with comorbid depression [37]. Older age and early implantation after the onset of epilepsy are associated with a better response; a longer epilepsy duration is associated with a lower response to therapy [38].

## 5. Technology Overview

### 5.1. Equipment

VNS equipment includes three categories of components: for surgical implantation; for programming use by a neurologist; and for patient use. Implanted components include a pulse generator, a lead receptacle (dual-pin or single-pin), circuitry with an antenna for telemetry to communicate with a programming wand, and a lead, which comes in two sizes to choose between, for a better fit based on nerve size (2 and 3 mmm inner helical diameters). These components come in a surgical kit that also includes a tunneler (i.e., a device to tunnel the lead subcutaneously between the neck and chest incision sites), an accessory pack for replacement components, and parts for intra-operative testing. Further details of surgical implantation and complications are discussed below.

After surgery, the patient receives a manual, identification cards, and two therapy magnets (wrist band and a belt-clip) for emergent use. The neurologist receives a programming wand and a programmer (a hand-held portable device), both used during patient visits to interrogate therapy settings and battery generator status, and to program the VNS when indicated. The programming wand is a hand-held device that receives and transmits data between the implanted generator and the programmer. It exists in three models, with the latest release in 2017 (Model 2000) as of this publication. The current model programmer is a tablet with touch screen and programming software that connects to the programming wand. Battery indicators vary depending on the implanted VNS model. The NeuroCybernetic Prosthesis (NCP) and M102 models only indicate near end of service (N EOS). Other models indicate the remaining battery power but show no warning signal for battery generators at more than 18% for M103/104, and more than 11% for M105-1000. The lack of replacement of a battery generator in a timely manner can lead to (1) losing the ability to communicate with the programming software and (2) a decreased therapy effect [39].

### 5.2. Technical Upgrades

**Figure 2:**
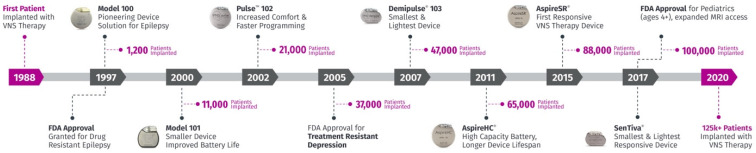
Vagus nerve stimulation (VNS) technology development timeline. Adapted with permission from LivaNova. VNS underwent several iterations to improve safety, tolerability, and therapy delivery. The graph shows different models and the number of patients implanted.

Since the first implant in 1988, VNS components underwent several upgrades targeted to improve safety, battery life duration, esthetics (smaller/lighter), and therapy (Figure 2) [40]. After FDA approval of the first NCP M100 model in 1997, a change from a dual pin to a single pin decreased the risk of lead communication problems in the models Demipulse M103 and Demipulse Duo (R) M104. Additional innovations provided smaller generators (Pulse TM M102R, M104) and higher capacity (AspireHC (R) M105, 36% longer lifespan). In 2015, Sensor technology (AspireSR (R) M106) provided a “sense and respond” capacity, introducing the first responsive closed-loop form of VNS therapy. The autostimulation mode detects rapid increases in heart rate that may be related to a seizure and responds with extra stimulation to try to abort the suspected seizure [41,42]. The most recent model, SenTiva M1000, combines the closed-loop autostimulation function in a smaller battery canister, and increased communication speed by four-fold for the better transfer of information to the programming wand [43]. Studies showed the merits of autostimulation, with better efficacy in adult and pediatric populations [44,45].

Other innovations include a wireless programming wand, remote titration capability, day/night programming that may benefit patients based on the timing of their seizures, and event detection. A more recent release, the Sentiva DuoTM M1000-D, allows patients with older generation dual-pins to access the latest innovations, similar to features provided by Sen Tiva M1000 [40]. Figure 2 illustrates the timeline of VNS upgrades.

## 6. Neuromodulation Programming

VNS dose adjustment programming determines how and when the VNS stimulates the vagus nerve to modulate brain activity [46]. Dose adjustment can be completed with an interrogation wand and a programmer; initial programming is typically scheduled two weeks after implantation, though some physicians opt to initiate low-current settings on the day of surgery. The features depend on the model, but all models typically include normal mode and magnet mode. Other features are autoStim mode (Model 106 and 1000), scheduled programming, and day–night programming features.

Neurologists typically start by programming low-current stimulation (0.5 mA), then incrementally increase the current by 0.125 to 0.25 mA at a time, depending on the model and output current, with a maximal output of 3.5 mA [21]. Titrating the current has typically required in-person programming, although the most recent model allows for remote scheduled programming, reducing the need for office visits.

The frequency is typically set at 20–30 Hz. Preclinical studies showed anticonvulsant benefits of 20 and 30 Hz frequencies, but more recent findings highlight mood benefits typically at 20 Hz. Therefore, 20 Hz is recommended for most recent generators [15]. The details for each model’s parameters are in the Physician’s Manual. For models NCP and M102, a specific warning recommends a minimum frequency of greater than 5 Hz, as, at 5 Hz or below, there is a risk of generating an electromagnetic trigger, leading to rapid battery consumption (hereinafter Physician’s Manual) (Section 1).

After applying a prediction model to data available from 12 clinical studies, Fahoum et al. demonstrated that output currents near 1.6125 mA and those programmed to a duty cycle near 17% (duty cycle is the percentage of time the generator is stimulating) are associated with the best response at 1 year [38]. Based on these findings, they speculate that patients programmed to higher settings may not benefit from a higher dose and that some patients will not respond to any setting [38]. For clinical use, a pulse width of 250 microseconds is recommended. A higher pulse width is associated with faster battery depletion and higher side effects [38,47]. The ASCEND trial has looked into titration strategies and has shown feasible titration to the recommended target current in 12 weeks or less [48]. Other scholars have demonstrated possible benefits from rapid cycling (OFF time ≤ 1.1 min and duty cycle less than 50%), predominantly in pediatric patients with drug-resistant epilepsy [49].

Recent studies have investigated predictors of treatment response by leveraging EEG network analysis and functional neuroimaging [50,51,52].

The therapy magnet that the patient receives can be used by a patient or caregiver to abort or decrease the intensity of a seizure. Upon noticing a seizure semiology, the patient or caregiver activates on-demand stimulation by passing the magnet over the generator. The patient can also temporarily stop stimulation by holding the magnet in place on the generator, or by affixing the magnet to the generator using tape or a wrap; stimulation restarts when the patient takes off the magnet. The patient may want to stop stimulation while getting used to new programming settings or use the magnet to trigger a mark in the VNS system (i.e., to mark a moment in time for later review). The neurologist can review this mark during a visit by interrogating magnet swipes. Newer generation closed-loop models can detect an increase in heart rate that may accompany an ictal phase and will prompt stimulation to try to abort the seizure (Model 106 and 1000). The sensitivity of the detection can be adjusted for heart rate changes from 20 to 70%. The higher the detection threshold, the better the accuracy for ictal tachycardia. In patients with activated tachycardia detection, it is important to deactivate this function before engaging in physical activity.

Other neuromodulation therapies, such as responsive neurostimulation and deep brain stimulation, are used in drug-resistant epilepsy. Recent studies have shown that combining neuromodulation therapies is safe and may contribute to synergic effects for better seizure control, in combination with responsive neurostimulation or deep brain stimulation [53,54,55,56].

## 7. Surgical Implantation and Complications

### 7.1. Implantation

The surgical implantation technique of VNS for epilepsy treatment was originally described by Reid et al. [57], and has recently been elegantly outlined by Giordano et al. [4]. A standardized procedure guide is also available from LivaNova, the only current manufacturer for an implantable VNS for the treatment of epilepsy [15]. During implantation, a cuff electrode is implanted around the left vagus nerve midway in the neck and connected to an implanted pulse generator placed just caudal to the clavicle. Due to the higher risk of bradycardia and asystole associated with stimulation of the sinoatrial node, innervated by the right vagus nerve, the left vagus nerve is used [58]. Although the left vagus nerve also has some cardiac branches that innervate the atrioventricular node by way of the recurrent laryngeal nerve, cardiac effects are thought to be less frequent.

All components for VNS implantation are provided in a kit from the manufacturer, including the following: generator, lead wire with two helical electrodes and a tethering anchor, and a disposable subcutaneous tunnelizer.

The surgery is performed under general anesthesia. A transverse incision is made at the midpoint of the sternocleidomastoid, from its medial border to just shy of midline. The carotid sheath is opened, and the vagus nerve is found deep, next to the common carotid artery and jugular vein. The diameter of the nerve is measured and either a two- or three-millimeter sized helical electrode is used. Electrodes must be placed inferior to cardiac branches to reduce the risk of cardiac complications. The tethering anchor should be secured first around the nerve and placed inferior to the positive electrode, which must be inferior to the negative electrode. A subcutaneous pocket caudal to the clavicle is made for the generator, and the lead is tunneled to this location. Once the lead is connected to the generator, the device is tested in the operative room prior to closure, using settings set by the manufacturer for each generator model. Those settings vary depending on whether the device output is set to 0 (new implant) or higher (battery change) (see Physician’s Manual p.52, Section 3.3.8.1, Table 26) [15]. The acceptable impedance range is 600 to 5300 ohms. A high impedance may be due to the following: a dry nerve, poor electrode contact, lead discontinuity, or a loose pin at the generator header [15]. Heart rate sensitivity is also measured when using a model that is to heart rate, indicated for patients who have tachycardia associated with their seizures. Once telemetry is completed, the electrode lead is secured to fascia in the neck, with a strain relief loop placed. The excess lead is coiled behind the generator, which is placed into the subcutaneous pocket and secured to deep fascia. Surgical wounds are then closed in the standard fashion.

Patients should be counseled on mild transient dysarthria and dysphagia after surgery due to the manipulation of the vagus nerve and from intubation. For this reason, some centers program the generator at the lowest setting (0.5 mA), whereas other centers leave the device off with a plan for close programming at the first post-operative visit. Generally, patients are discharged from the hospital on the day of surgery or the following morning.

### 7.2. Revision/Removal

VNS revision accounts for half of cases (46% of VNS-implanted patients), often for battery replacement or device malfunction [59].

The generator requires replacement at regular intervals based on the amount of stimulation delivered, which depends both on the programmed baseline settings as well as additional triggers for stimulation by magnet swipe or tachycardia. When a generator returns a near end of service battery life indicator to a programmer (see Physician Manual; Section 2 on N EOS indicator variability between models) [15], generator replacement should be scheduled. This can be achieved through an outpatient procedure under moderate sedation or general anesthesia, depending on the patient’s ability to tolerate surgery. Only the subcutaneous pocket for the generator needs to be opened for device replacement, and the electrode does not need to be manipulated.

In cases of superficial infection, if the device is functioning, conservative treatment with antibiotics should be attempted prior to surgical revision. For deep-seated infection or device exposure (i.e., skin erosion), complete removal including the helical coil is recommended [4,59,60]. This can be achieved using sharp and blunt dissection of helical coils or can be combined with low-voltage cautery dissection [61]. After infection is treated with antibiotics, re-implantation can be performed. The surgical technique for VNS revision is detailed in a comprehensive review by Giordano et al. [4].

In cases of device malfunction—such as out-of-range impedances, telemetry communication errors, or unexpected stimulation side effects—surgery is generally required. The integrity of the lead and the connection between the lead and generator should first be investigated before committing to a complete revision. When the lead cannot be safely explanted, due to adhesions to the vagus nerve, the helical coils remain and the lead is cut with <2 cm of lead remaining, to conditionally permit future MRI [15]. A new electrode can then be placed more proximally along the nerve if required.

### 7.3. Expected Side Effects of Therapy

The side effects of VNS therapy are usually related to the stimulation itself. Laryngopharyngeal dysfunction is the most common late complication, affecting 66% of cases with hoarseness, dyspnea, and coughing. Laryngopharyngeal dysfunction is caused by stimulation of the neighboring branches from the inferior (recurrent) laryngeal nerve, typically related to stimulation frequencies, and is often transient [62]. Permanent vocal cord paralysis is rare. Other delayed complications include arrhythmias (bradycardia, asystole), obstructive sleep apnea, hiccups with anecdotal cases of refractory hiccups, due to proximity of the vagus nerve to the phrenic nerve, tonsillar pain (mimicking glossopharyngeal neuralgia), and drooling and hyperactivity in children on rare occurrences [4,63].

## 8. Complications

Early complications of VNS are generally considered to be related to the surgical procedure. During lead impedance testing, there is a risk of bradycardia and asystole (1/1000 cases) [64]. Other surgical risks and complications include peritracheal hematoma, infection (3–8%), injury to vagus nerve fibers or to its blood supply, leading to vocal cord paralysis, or vocal cord damage due to prolonged endotracheal intubation [62,65]. This manifests with hoarseness, dyspnea, and dysphagia. However, the left vocal cord paralysis is often transient, and symptoms resolve within a few months [4].

Delayed complications of VNS are usually device-related. These include wound dehiscence and infection. Though rare, late injury of the vagus nerve with permanent vocal cord paralysis has been described, including the following: blunt injury to the nerve, stretching of the nerve because of insufficient strain loop relief during implantation, and “twiddler’s syndrome” (lead retraction leading up to lead fracture) in obese and cognitively impaired patients [4].

VNS device malfunction can be caused by intrinsic microlesions, visible lead fractures, short circuits, and electrode coil dislocation [66]. These were more common in the oldest VNS system models (M301, M302). Device malfunction rates have decreased in newer VNS models.

## 9. Other Considerations

SUDEP rates are higher in people with drug-resistant epilepsy compared to those with controlled epilepsy and the general population [67,68]. Therefore, any therapy that may lower seizure frequency or severity could potentially decrease risks for SUDEP. Large retrospective studies using patient registries found a lower rate of SUDEP two years after implantation [69,70]. Moreover, others demonstrated lower risk for SUDEP by showing a reduction in T-wave alternans (TWA), a surrogate marker of risk for SUDEP, after VNS therapy in DRE [71].

**Figure 3:**
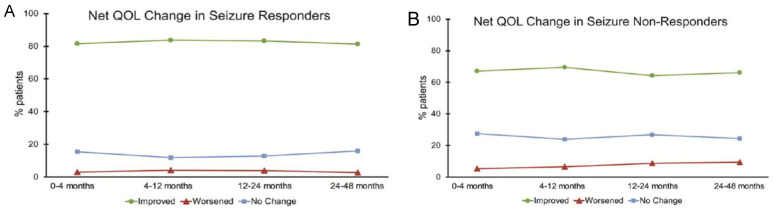
Quality of life is more likely to improve in patients who respond to VNS therapy. Adapted from Englot et al., 2017. License 5773240606721 [74].

Seizure freedom is the most important predictor of quality of life (QOL) in epilepsy [72,73]. In patients implanted with VNS, Englot et al. found improvement in several physician-reported QOL metrics (Figure 3) that are more likely to occur in patients who were respondent to VNS [74].

### 9.1. VNS and Neurological Disorders

Separate from increased mortality, patients with epilepsy have increased neuropsychiatric disorders, most notably depression and anxiety, and heterogeneous cognitive complaints.

#### 9.1.1. Depression

Major depression is currently underdiagnosed in epilepsy. It affects 13 to 38% of people with epilepsy [75,76]. VNS efficacy on epilepsy comorbidities has been demonstrated since 2000, with evidence of its efficacy on mood and comorbid depression and anxiety [77,78,79]. This is supported by animal and human neurochemical studies that show variation in brain monoamines (serotonin, norepinephrine, gamma aminobutyric acid (GABA), and glutamate) and neuroimaging research (positron emission tomography, functional MRI) [80,81,82]. Functional neuroimaging studies have found increased signal, using blood oxygenation level dependent (BOLD) functional MRI, better at 250 and 500 μs pulse width (compared to 130 μs) [83]. Currently, the most commonly used settings for treatment-resistant depression are as follows: 500 μs pulse width, 20 Hz frequency, and 30 s/5 min on/off time [84].

#### 9.1.2. Motor Rehabilitation

VNS application to neuronal plasticity is still an area under investigation. To our knowledge, a few clinical trials in stroke patients were conducted. These studies have shown a small positive effect in stroke patients with implanted VNS compared to controls who received standard physical therapy [85,86].

While a more recent randomized, controlled trial (sham group was implanted without active stimulation) has found better improvement in upper extremity motor response after 6 weeks and 60 days of the physical therapy combined with VNS [87]. These findings were supported by a more recent study, in patients implanted after stroke and treated with acute rehabilitation [88].

Other research has investigated therapeutic effect of implanted VNS on cognition in people with drug-resistant epilepsy, with variable results [89,90,91,92,93]. A comprehensive review of neuropsychiatric co-morbidities of epilepsy and treatments including VNS can be found in the following reference [92].

## 10. Conclusions

In this review, we have given an overview of the role of VNS, specifically in patients with epilepsy. Thoughtful patient selection in regard to seizure type, current anti-seizure regimen, comorbid neuropsychiatric symptoms, and risk for SUDEP can lead to the beneficial use of VNS.

VNS’s role continues to evolve. In epilepsy treatment, there are investigations of transcutaneous VNS (t-VNS), a noninvasive option that targets the cervical or the auricular branch at the surface of the skin. Beyond the scope of epilepsy, VNS has approval for use in depression and in cluster headache. Ongoing research will elucidate the neurophysiological underpinnings of VNS, broadening its impact on patients with and without epilepsy. The overarching effect of VNS on local brain and on the inhibition of systemic inflammation highlights the importance of future research on mechanisms underlying VNS efficacy, to guide patient selection in epilepsy and depression, and to discover novel therapies for other neurological disorders.
